# Cascaded Deep Learning Frameworks in Contribution to the Detection of Parkinson’s Disease

**DOI:** 10.3390/bioengineering9030116

**Published:** 2022-03-12

**Authors:** Nalini Chintalapudi, Gopi Battineni, Mohmmad Amran Hossain, Francesco Amenta

**Affiliations:** Centre of Clinical Research, School of Medicinal and Health Products Sciences, University of Camerino, 62032 Camerino, Italy; gopi.battineni@unicam.it (G.B.); mohammad.hossain@unicam.it (M.A.H.); francesco.amenta@unicam.it (F.A.)

**Keywords:** Parkinson’s disease, deep learning, neural networks, model fitting, early detection

## Abstract

Parkinson’s disease (PD) is a progressive neurodegenerative disorder characterized by motor impairment, as well as tremors, stiffness, and rigidity. Besides the typical motor symptomatology, some Parkinsonians experience non-motor symptoms such as hyposmia, constipation, urinary dysfunction, orthostatic hypotension, memory loss, depression, pain, and sleep disturbances. The correct diagnosis of PD cannot be easy since there is no standard objective approach to it. After the incorporation of machine learning (ML) algorithms in medical diagnoses, the accuracy of disease predictions has improved. In this work, we have used three deep-learning-type cascaded neural network models based on the audial voice features of PD patients, called Recurrent Neural Networks (RNN), Multilayer Perception (MLP), and Long Short-Term Memory (LSTM), to estimate the accuracy of PD diagnosis. A performance comparison between the three models was performed on a sample of the subjects’ voice biomarkers. Experimental outcomes suggested that the LSTM model outperforms others with 99% accuracy. This study has also presented loss function curves on the relevance of good-fitting models to the detection of neurodegenerative diseases such as PD.

## 1. Introduction

Parkinson’s disease (PD) is a progressive neurodegenerative disorder characterized by three main cardinal motor symptoms, namely, bradykinesia (slowness of movement), rigidity, and resting tremors. PD represents the second most common neurodegenerative disorder after Alzheimer’s disease [1,2]. Besides the characteristic motor symptoms, PD presents several non-motor symptoms that contribute to increases in the overall disease burden to different extents. The non-motor symptoms of PD include hyposmia, constipation, urinary dysfunction, orthostatic hypotension, memory loss, depression, pain, and sleep disturbances [2]. The motor signs of PD are linked to nigral degeneration and striatal dopamine depletion, whereas the non-motor symptoms are probably associated with the neurodegeneration of other brain structures [1]. The cognitive impairment of PD represents one of the most relevant non-motor correlates of this disorder, and may affect memory, thinking, learning capacity, language, judgment, behavior, and daily living activities [1].

Today, the clinical diagnosis of PD is primarily based on the presence of motor symptoms, although the neurodegeneration responsible for PD starts long before the onset of motor symptoms, and non-motor changes can occur during the earlier phases of neurodegeneration [3]. On the other hand, diagnostic misclassification of PD is not uncommon, with error rates ranging from 15% to 24% reported by different studies. It is thought that, even using very accurate diagnostic criteria, approximately 10% of people diagnosed with PD by neurologists suffer from different pathologies [2,4].

To overcome these difficulties, an emerging field of machine learning (ML) that is drawing considerable attention is deep learning. Deep learning is a subfield of ML, which uses many layers of neural networks and learns features through the hierarchical learning process [5]. The traditional ML algorithm normally carries relevant features that act as model input; however, the deep learning algorithm can work on raw data and derive the features itself. Deep learning’s methods for classification or prediction are applied in various fields including Natural Language Processing (NLP) and computer vision. Initially, most deep learning applications in neuroscience focus only on the “downstream” of the detection and segmentation of anatomical structures and scratches, such as hemorrhage, stroke, lacunes, microbleeds, metastases, aneurysms, primary brain tumors, and nervous tissue hyperintensities [6]. On the “upstream” side, we now realize that Artificial Intelligence (AI) has other innovative applications in various technical aspects of medical imaging.

Various approaches to image generation and image enhancement through in-depth learning have recently been proposed, including normalizing or harmonizing images, removing patterns, improving quality, shortening the duration of image studies, and reducing radiation and contrast doses. There are two sorts of neural networks mostly used: Convolutional Neural Networks (CNNs) and Recurrent Neural Networks (RNNs). CNNs are frequently used for the tasks of image recognition and classification, whereas RNNs are recommended for building up a sequential representation of data over time. In [7], the authors presented deep-learning-based approaches to diagnosing Parkinson’s disease among twenty individuals, presenting a thirteen-layer CNN model that showed 88.25% accuracy. Farhan et al. [8] proposed a deep learning model for PD diagnosis by using medical images. They developed CNN-based models with 10-fold cross-validation and produced a high accuracy of 99.34%. Similarly, the Long Short-Term Memory (LSTM) network model for automatic and non-invasive PD diagnosis and multi-class classification has achieved an average accuracy of 96.6% [9]. A novel Deep Neural Network (DNN) classifier for the automatic detection of PD with a 1D-CNN architecture is proposed in [10], and presented a 98.7% accuracy. Another study of comparative ML approaches to assist in the diagnosis of PD used MRIs for men and women individually, with an accuracy of 99.01% and 96.97%, respectively [11].

Deep learning algorithms have been applied to analyses of raw neuroimaging data, even in the lack of feature selection processes, as well as to PD diagnostic classifications. MRI imaging that uses these deep learning algorithms can be more accessible from many perspectives, including cost, patient safety, and patient satisfaction. CNNs process images from the base. The neurons that are located earlier within the network are in control of examining the small windows of pixels and detecting simple, small features such as edges and corners. These outputs are then sent into neurons within the intermediate layers, which search for larger MRI features such as cysts, tumors, bleeding, swelling, developmental and structural abnormalities, infections, inflammatory conditions, or problems with the blood vessels. This second set of outputs is used to form a due process as to whether the image contains features of dementia. RNNs can store previous network outputs and use those as inputs for future computations. Information from these steps helps the network to make smarter and more accurate decisions. LSTMs can add more future uses to RNNs by recollecting important information and forgetting irrelevant values.

Therefore, in this work, we explore the three cascaded deep learning techniques, namely, multi-layer perception (MLP), RNN, and LSTM, in the classification of the voice biomarkers for PD diagnosis. For dealing with imbalances in the dataset, minority class oversampling was applied. Furthermore, the performance of each model was validated in terms of model accuracy, loss, precision, recall, and F1 score.

## 2. Methods

### 2.1. Data Collection

We consider a Parkinson’s disease patient dataset consisting of voice biomarkers from the UCI machine learning repository [12]. The dataset has a combination of 23 people with Parkinson’s disease and 31 biomedical voice measurements. The columns represent a special voice measurement and relate to a voice recording of each subject that attended at least six speech sessions. Subject demographics including sex, age, and PD status are presented in Table 1.

The main aim is to classify the 31 subjects as either healthy or having PD according to their biomedical voice measurements. To do that, speech data were used in this study. The variable named “status” was set to healthy and the PD variable to values of 0 and 1, individually. This is the target variable or dependent value for our model to classify. Others features: average vocal fundamental frequency (MDVP: Fo (Hz)), maximum vocal fundamental frequency (MDVP: Fhi (Hz)), minimum vocal fundamental frequency (MDVP: Flo (Hz)), several measures of the variation in fundamental frequency (MDVP: Jitter (%), MDVP: Jitter (Abs), MDVP: RAP, MDVP: PPQ, Jitter: DDP), several measures of the variation in amplitude (MDVP: Shimmer, MDVP: Shimmer (dB), Shimmer: APQ3, Shimmer: APQ5, MDVP: APQ, Shimmer: DDA), two measures of the ratio of noise to tonal components in the voice (NHR, HNR), two nonlinear dynamical complexity measures (D2), signal fractal scaling exponents (DFA). Three nonlinear measures of fundamental frequency variation (spread1, spread2, PPE) are independent values [13].

### 2.2. Data Pre-Processing and Visualization

We used a Pandas DataFrame to visualize the data set. Data info such as data type and the null value of any column was checked by using the info function. The data preprocessing step involved two methods, including the handling of missing data and data normalization. In this given PD dataset, there were no missing value for any columns. We did not handle null/missing values in this experiment. The next step was data normalization. Here, we normalized the voice features using min-max normalization, which involves the method of rescaling the data to [0, 1]. This standardization was used feature-wise and helps to improve the numerical stability of the model.

The dataset contains information on 195 sustained vowel phonations with binary status classification (healthy: 0 and PD: 1), with the “status” of binary values 0 and 1 corresponding to 48 and 147 individual records, respectively. To deal with the imbalance, we used the Synthetic Minority Oversampling Technique (SMOTE) resampling technique [14]. The motivation behind this study is the classification of voice signals of PD subjects based on status of PD and health (target variable). Here, we are not doing patient sampling, but instead sampling of the voice biomarkers variable data was done. The analysis conducted data resampling by SMOTE to make a balanced dataset using minority class oversampling.

**Figure 1:**
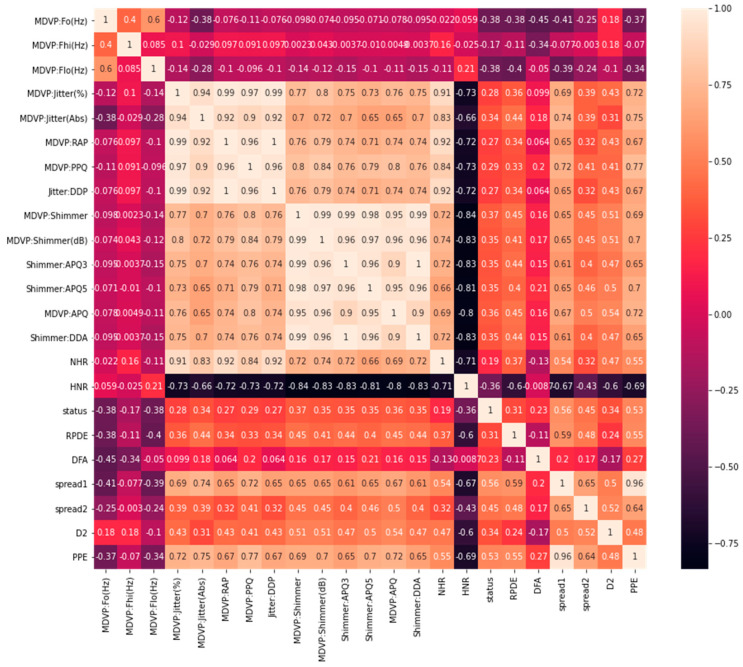
Correlation heatmaps.

On the other hand, the correlation matrix [15] can be useful in the visualization of variables highly correlated with PD status classification. This helps to visualize the summarization of data and understand the relationship between the independent variables and the targeted outcomes. The correlation between different columns, and especially with our target column “status”, is depicted in Figure 1. There are only 24 variables, including the target value. The first column “name” is an object and not correlated with our model, so we excluded the name variable. We used 23 variables to remain in our model input, except “status”, which was used to predict the results.

### 2.3. Description of Methods

In this paper, we applied ML algorithms to understand the link between PD diagnosis and model building for early disease diagnosis. Performance analysis of the three different deep learning algorithms multi-Layer perception (MLP), Recurrent Neural Networks (RNN), and the Long Short-Term Memory (LSTM) model were studied in the detection of PD detection.

#### 2.3.1. Multi-Layer Perception

**Figure 2:**
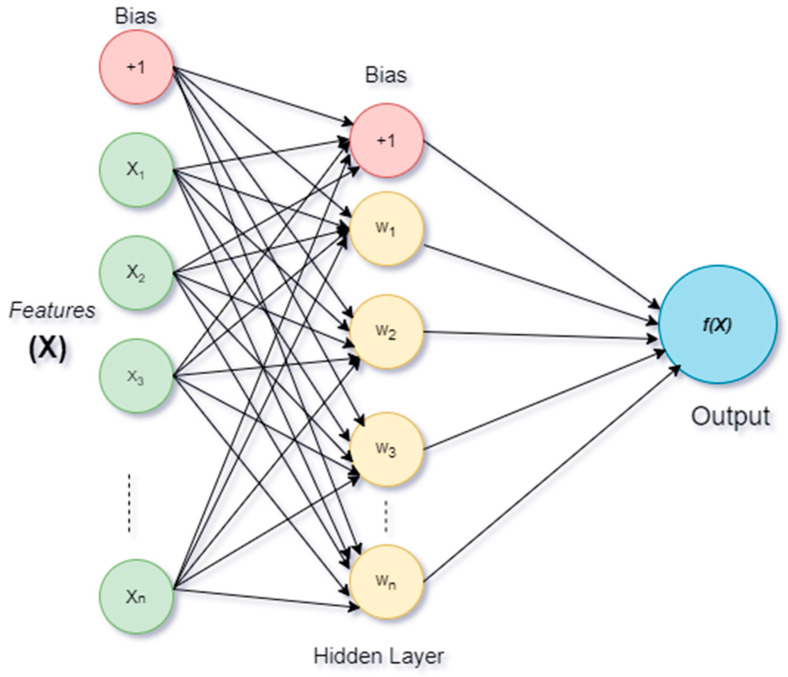
MLP architecture with one layer.

MLP is a neural network algorithm for supervised learning that can learn nonlinear function approximators for regression and classification from a given set of features $X=x_{1},x_{2},\ldots ,x_{n}$ and a target y. Here can be one or more of the hidden layers (nonlinear) between the input and output layers. MLP algorithms train on the dataset to learn function $f\left(.\right):\;R_{n}\to R_{o}$ with *n* number of input dimensions and o number of output dimensions. Figure 2 shows MLP for a scaled output with one hidden layer.

The leftmost layer is the input layer, with a set of neurons $\left\{x_{i}|x_{1,}x_{2},\ldots ,x_{n}\right\}$ which represent the features of the input. Those input features transform in the hidden layer by a weighted linear summation $w_{1}x_{1}+w_{2}x_{2}+\ldots +w_{n}x_{n}$, followed by a nonlinear activation function $\left(.\right):\;R_{n}\to R_{o}$. The output layer receives values from the previously hidden layer and converts them into outputs.

Mathematically, for a given training set $\left(x_{1},y_{1}\right),\left(x_{2},y_{2}\right),\ldots ,\left(x_{n},y_{n}\right)$ where $x_{i}\in R_{n}$ and $y_{i}\in \left\{0,1\right\}$, a hidden layer or a hidden neuron MLP layer learns from the function $f\left(x\right)=W_{2}g\left(W_{1}^{T}x+b_{1}\right)+b_{2}$, where $W_{1}\in R_{n}\;\mathrm{and}\;W_{2},b_{1},b_{2}\in R$ are model parameters. *W*_1_ represents the weights of the input layer and *W*_2_ is the weights of the hidden layer. The bias added to the hidden and output layer is represented as *b*_1_ and *b*_2_ , respectively. The activation function $g\left(.\right):\;R_{n}\to R_{o}$ is set as the default for the hyperbolic tangent, and is given as

(1) $$g\left(z\right)=\frac{e^{z}\;-\;e^{-z}}{e^{z}+e^{-z}}$$

To obtain output values between zero and one, binary classification f(x) passes through the logistic function g(z)=1(1+e−z), also called a sigmoid function. If the output values of the samples are larger or equal to a threshold (set to 0.5), are assigned to the positive class and the rest to the negative class.

#### 2.3.2. Recurrent Neural Networks (RNN)

**Figure 3:**
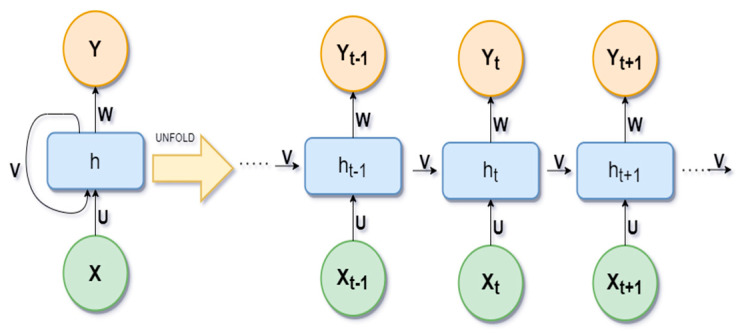
Unfolding RNN architecture.

Recurrent neural networks (RNNs) are a class of neural networks. In RNNs, the connection between nodes forms a directed graph along a temporal sequence. It allows for the use of their internal state (memory) to store previous outputs to be used as inputs for hidden states. The basic idea of unfolding RNN architecture is presented in Figure 3.

Here, X is the input variables, h is the hidden layer vector, and Y is the output layer vector. W and U are parameters. The loss function ℒ of RNN is defined as $ℒ\left(ˆy,y\right)=\sum_{t=1}^{t_{y}}ℒ\left(ˆy^{t},y^{t}\right)$. The most common activation functions used in RRN are Sigmoid, Tanh, and RELU. The sigmoid activation function is used for binary classification with a range of (0, 1). The tanh activation function is like the sigmoid, but the range is different (−1, 1). RELU produces an output of zero if the input is less than zero, otherwise it produces x, and can be represented as:

(2) $$f\left(x\right)=\{\begin{array}{cc} 0,\;\; & if\;x<0\\ x,\;\; & Otherwise \end{array}$$

#### 2.3.3. Long Short-Term Memory (LSTM)

Long Short-Term memory (LSTM) is a neural network algorithm for deep learning applications augmented by a recurrent neural network. LSTM is a special kind of RNN that is designed to avoid long-term dependency problems. Its default behavior is remembering information for long periods. Like all other recurrent neural networks LSTM takes the form of a chain of repeating modules of a neural network.

**Figure 4:**
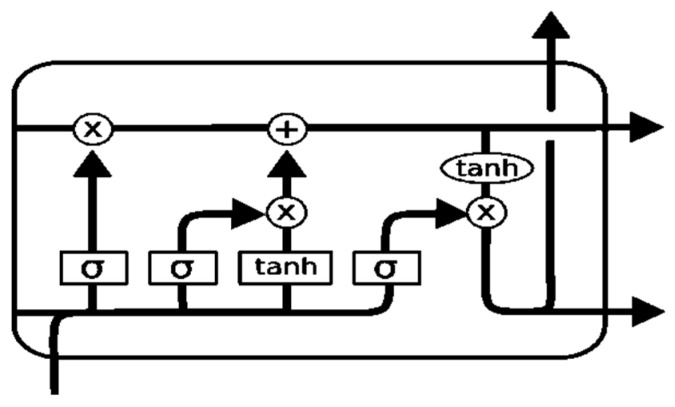
LSTM architecture.

An LSTM has three gates to protect and control the cell state. It could add or remove information, which is carefully controlled at the gate, as a way to optionally let information pass. The first step or gate is called the forgotten gate level, where the sigmoid decides whether any information is kept or discarded. The gates are composed of a sigmoid layer and a pointwise multiplication operation. The output of the sigmoid layer is between 0 and 1, where 1 means the information needs to be stored for the next use and 0 means it does not need to store. The next step, consisting of two parts, is to decide what new information is going to be stored in the cell state. First, the input gate layer consists of a sigmoid that decides which value will be updated. A vector of new values is created by the tanh layer, which could be added to the state. In the final step, both are combined to update the state. Finally, the output will be filtered and based on the cell state. The first sigmoid layer decides which part will be the output and then passes it through the tanh layers. The tanh layers’ value will multiply with an output of the sigmoid gate to obtain the desired output. According to [16], every LSTM layer expects a 3D array of data during model fitting when predictions were performed, although specific array dimensions contain a single array value. Therefore, we need to reshape our input dataset into 3D. The basic architecture of the LSTM model is presented in Figure 4.

### 2.4. Experiments

The experiments were conducted in three cascaded deep neural networks that encoded the hidden information inside the audio features, and interpreted the PD elements that trigger the audio features to complete patient classification. The presented deep neural networks were designed in this experiment by using the Keras and TensorFlow libraries. A linear unit activation function (ReLU) was used through the layer. The ReLU allowed us to modify default parameters and to use non-zero thresholds. This function also changed the max activation value and used a non-zero multiple for the input value which was lower than the threshold value. A sigmoid activation function, sigmoid(x)=1/(1+exp(−x)), was used through the output layer. The sigmoid function was applied to the small values and it returned a value close to zero, and approached one for the large value.

For MLP, we used 24 dense layers as inputs and “ReLU” as the activation function. For the hidden layer, we used 32 dense layers and the ReLU activation function. We used the sigmoid activation function on the single dense-layer output. In RNN, we used 32 flattened dense layers as the input layer and 16 dense layers for the hidden layer. This model also used the ReLU activation function. The sigmoid activation function is used for obtaining output from a range of 0 to 1. In the LSTM model, we maintained 100 input and hidden layers, and the activation function ReLU is the same as other models. Here we also added a dropout of (0.5). We obtained the output with the sigmoid function.

### 2.5. Implementation and Validation Details

We split our dataset for training and testing so that we could measure the accuracy, loss, precision, recall, and F1-score of these three ML algorithms in PD detection. This unbalanced dataset has been split stratified by utilizing class labels to determine the number of samples from each target class in each subset. In a stratified style, each subset contains approximately the same number of samples from each target class as the complete dataset. The data was split into 67% training data and 33% testing data.

The training dataset is used to train the ML model and the test dataset is used to predict the cases with and without PD with the trained model. We train deep learning models in small batches of 16 samples with cross-entropy as a loss, by applying the stochastic gradient descent (SGD) algorithm.

The splitting is repeated for a maximum of 100 epochs to avoid infinity loop iterations. We stopped at the 30th iteration to prevent the model from overfitting. To evaluate each model, we tested different numbers of epochs to pick the best epoch value; batch size = 1, since the dataset was not large, 131 (67% of 195). For the loss function, we used binary cross-entropy [17] and an Adam optimizer [18]. The reason behind the selection of cross-entropy is it was the simplest approach to the probability measurement of the model. It can be useful because of its capability in model description in the likelihood of error functions for every datapoint, and in describing predicted outcome compared with the actual outcome. We reported model performance in terms of model accuracy and loss.

### 2.6. Performance Metrics

Accuracy and loss metrics are used to calculate the model performance. These metrics create two local variables, “total” and “count”, to calculate how often the predictions are equal with the labels. These local variables match with the target prediction (y_pred) and true (y_true) classes to compute frequency, which is returned as a binary accuracy. The model accuracy can be defined as the percentage of correct predictions, or it can also be written as the ratio of correct predictions to total predictions, such as:

(3) $$\mathrm{Accuracy}=\frac{\mathrm{TP}+\mathrm{TN}\;}{\mathrm{TP}+\mathrm{TN}+\mathrm{FP}+\mathrm{FN}\;}$$

where TP is true positives; TN, true negatives; FP, false positives; and FN, false negatives.

As mentioned, all models used the binary cross-entropy loss function, calculating the cross-entropy loss between the predicted classes (y_pred) and the true classes (y_true; either 0 or 1) in the model prediction, either represented as a logit or probability. The loss function is defined as

(4) Loss=True classes−Predicted classes

Other performance metrics such as precision, recall, and F1 score also define the model performance. The definition of those metrics is given below.

Precision is the percentage of true positives and is mathematically represented as

(5) $$\mathrm{Precision}=\frac{\mathrm{TP}}{\mathrm{TP}+\mathrm{FP}}$$

Recall is the calculation of a percentage of true predicted positives among total positives, and is also called true positive rate (TPR)

(6) $$\mathrm{Recall}=\frac{\mathrm{TP}}{\mathrm{TP}+\mathrm{FN}}$$

F1 score is the harmonic mean of precision and recall and considers both false positives and false negatives. This metric performed well in imbalanced datasets.

(7) $$F1\;\mathrm{score}=\frac{2\times \left(\mathrm{Precision}\times \mathrm{Recall}\right)}{\mathrm{Precision}+\mathrm{Recall}}$$

## 3. Results

**Figure 5:**
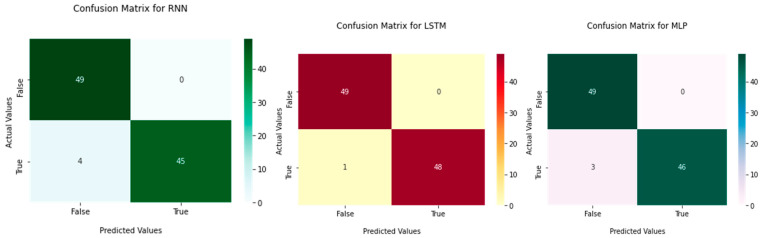
Confusion matrix outcomes for three deep learning models.

Our three developed models classify the healthy and PD patients according to these independent values. To calculate the performance of the adopted models, confusion matrices were considered, and Figure 5 presents the confusion matrix outcomes for these models.

Table 2 provides an overview of experimental outcomes; from this, we obtain our model’s training and testing accuracy, as well as loss score, and we can easily identify the best model from it.

**Figure 6:**
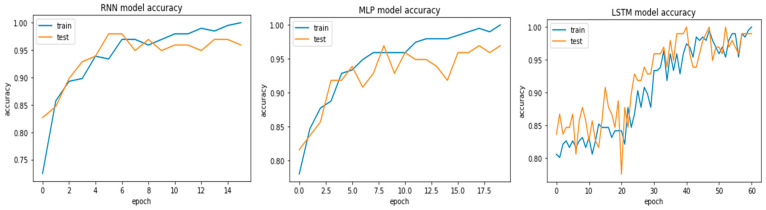
Testing and training accuracy of RNN (**left**), MLP (**middle**), and LSTM (**right**).

Figure 6 represents the accuracy curves of both the training and testing datasets. The testing dataset accuracy produced almost 98% accuracy with the LSTM model and 100% accuracy on the training datasets. RN and MLP generated 95.91% and 96.93% accuracy in testing, respectively, and both generated a 99.48% classification accuracy on the training datasets.Table 2 shows the detailed result of those three ML models with test and training accuracy, loss, precision score, recall score, and F1-socre.

**Figure 7:**
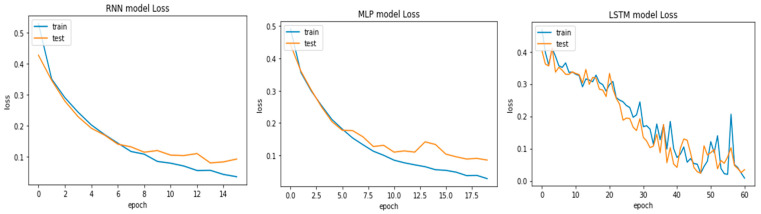
Testing and training loss of RNN (**left**), MLP (**middle**), and LSTM (**right**).

On the other hand, model fitting is a parameter that explains how well an ML model is generalized according to the same data on which training has been completed. The goal of good fit for the learning algorithm exists between the underfitting and overfitting of the model. The model’s good fit is recognized by a training and testing loss that reduces to the stability point with a small gap between both curves [19]. Similar to accuracy curves, we present the loss curves for the three adopted models in Figure 7. Observing them, the plots of the testing and training losses decrease to the stability point and have a small gap with the training loss, which proves that the models are well fitted with the dataset. The lowest training (3.5%) and testing (0.35%) loss was produced by the LSTM model, followed by the MLP and RNN models. Through these experiments, it is concluded that the LSTM performed better in the classification of voice signals.

## 4. Discussion

The current work presented cascaded deep learning frameworks for the classification of voice markers in PD diagnosis. Different data pre-processing techniques were applied before training of three deep-learning-based cascaded neural networking models. The results demonstrated that LSTM outperforms the other two models, MLP and RNN, respectively.

There is still no particular diagnostic process for PD detection, but there are different diagnostic tests and symptoms used in combination for diagnosis. Various biomarkers have been investigated by scientists for early PD identification. Current treatments improve PD symptoms without halting or slowing the disease process. The early detection of PD with better accuracy is important because of its ability to offer critical information to slow down disease progression. For many years, different data-driven methods were developed to advance PD detection. Compared to PD detection modeling techniques where the analytical model is a prerequisite, in data-driven methods only accessibility to historical data is required.

Deep learning techniques have the highest probability in the detection of PD. In recent times, deep-learning-type neural networks have emerged as a prominent source for research in the diagnosis of PD, both in industrial and institutional applications [20]. Due to their data-driven modeling techniques, neural networks have brought a paradigm shift to the process of analyzing relevant data in PD biomarkers. ML-based PD diagnosis with feature selection and classification modeling, and support vector machines (SVM) combined with recursive feature elimination, showed the highest accuracy of 93.84%, with a smaller number of voice biomarkers for PD [21]. Three different neural networks, namely RNN, MLP, and LSTM, were used in this study and showed different accuracy values for each classifier. The LSTM classifier outperforms the other two models and produced a maximum classification accuracy of almost 99%, as well as the best F1-score (98.96%), precision score (100%) and recall score (97.95%).

The present investigation has attempted to early prediction of the patient condition at the different disease severity levels over time. Previous studies proposed a model for predicting and diagnosing PD severity based on biomedical voice features. For instance, it was reported in [22] that Gaussian classification with autonomous relevance estimation has a 96.92% accuracy, which outperforms SVM and decision tree ensemble learning. It also proved that deep learning models can outperform ML models in the early detection of PD [23]. Another study proposed a deep learning-based neural network architecture for PD severity prediction [24].

However, this work is different from the previous literature on neural network implementation because of its simple classification and data normalization techniques. Maximum classification accuracy in terms of the AUC parameter was achieved, and using the K-fold validation technique helped very much in testing the model’s performance. When compared to MRI or motion-based diagnosis methods, obtaining voice biomarkers are both easy and cheap. These study outcomes suggest that LSTM with SMOTE oversampling can produce the best classification accuracy in PD subject classification. Our proposed models can be helpful neurological studies for disease diagnosis using biomarkers datasets.

Despite demonstrating the highest subject classification accuracy, this study has some limitations. Firstly, the low subject sample can hinder the outcome assumptions with respect to global PD patients. These advanced cascaded deep learning models can help in the accurate classification of PD subjects based on vocal abnormalities and others, but not confirm disease diagnosis. There is a need for further studies with consideration for confirmation and better sample characterization.

## 5. Conclusions

By using non-invasive voice biomarkers as features of automatic ML architectures, PD diagnosis and prediction are possible. In this paper, we compared the performances of three cascaded deep learning models for PD diagnosis using voice signals. Because of the use of SMOTE sampling in the assessment of neurological data to assess patient conditions, the experiments produced promising results. The proposed deep learning LSTM cascaded model showed excellent classification accuracy (~99%). LSTM models are desirable for learning of nonlinear and linear features from a PD dataset without requiring handcrafted feature-extraction techniques. Due to the higher accuracy of these models with simple audio features containing spoken words, LSTM can help in the accurate classification of PD subjects and the validation of future diagnostic approaches.

## Figures and Tables

**Table 1:** Subject demographics.

Subject Code	Age	Sex	Status	Years of Diagnosis
S01	78	M	PD	0
S02	60	M	PD	4
S04	70	M	PD	5.5
S05	72	F	PD	8
S06	63	F	PD	28
S07	46	F	Healthy	N/A
S08	48	F	PD	2
S10	46	F	Healthy	N/A
S13	61	M	Healthy	N/A
S16	62	M	PD	14
S17	64	F	Healthy	N/A
S18	61	M	PD	11
S19	73	M	PD	7
S20	70	M	PD	1
S21	81	F	PD	5
S22	60	M	PD	4.5
S24	73	M	PD	1
S25	74	M	PD	23
S26	53	F	PD	1.2
S27	72	M	PD	15
S31	-	-	PD	-
S32	50	M	PD	4
S33	68	M	PD	3
S34	79	F	PD	0.25
S35	85	F	PD	7
S37	76	M	PD	5
S39	64	M	PD	2
S42	66	F	Healthy	N/A
S43	62	M	Healthy	N/A
S44	67	M	PD	1
S49	69	M	Healthy	N/A
S50	66	F	Healthy	N/A

**Table 2:** Performance metrics of training and testing datasets with three neural network models.

Model	Test Accuracy	Test Loss	Training Accuracy	Training Loss	Precision	Recall	F1 Score
MLP	96.93	8.55	99.48	2.50	100	93.87	96.84
RNN	95.91	9.20	99.48	3.01	100	91.83	95.74
LSTM	98.97	3.46	100	0.34	100	97.95	98.96

## Data Availability

Not applicable.

## References

[1] Aarsland D., Batzu L., Halliday G.M., Geurtsen G.J., Ballard C., Chaudhuri K.R., Weintraub D. (2021). Parkinson disease-associated cognitive impairment. Nat. Rev. Dis. Prim..

[2] Tolosa E., Garrido A., Scholz S.W., Poewe W. (2021). Challenges in the diagnosis of Parkinson’s disease. Lancet Neurol..

[3] Gonzalez-Latapi P., Bayram E., Litvan I., Marras C. (2021). Cognitive Impairment in Parkinson’s Disease: Epidemiology, Clinical Profile, Protective and Risk Factors. Behav. Sci..

[4] Rizzo G., Copetti M., Arcuti S., Martino D., Fontana A., Logroscino G. (2016). Accuracy of clinical diagnosis of Parkinson disease. Neurology.

[5] Schmidt J., Marques M.R.G., Botti S., Marques M.A.L. (2019). Recent advances and applications of machine learning in solid-state materials science. NPJ Comput. Mater..

[6] Chan H.-P., Samala R.K., Hadjiiski L.M., Zhou C. (2020). Deep Learning in Medical Image Analysis. Prediabetes.

[7] Oh S.L., Hagiwara Y., Raghavendra U., Yuvaraj R., Arunkumar N., Murugappan M., Acharya U.R. (2018). A deep learning approach for Parkinson’s disease diagnosis from EEG signals. Neural Comput. Appl..

[8] Mohammed F., He X., Lin Y. (2020). Retracted: An easy-to-use deep-learning model for highly accurate diagnosis of Parkinson’s disease using SPECT images. Comput. Med. Imaging Graph..

[9] Balaji E., Brindha D., Elumalai V.K., Vikrama R. (2021). Automatic and non-invasive Parkinson’s disease diagnosis and severity rating using LSTM network. Appl. Soft Comput..

[10] El Maachi I., Bilodeau G.-A., Bouachir W. (2019). Deep 1D-Convnet for accurate Parkinson disease detection and severity prediction from gait. Expert Syst. Appl..

[11] Solana-Lavalle G., Rosas-Romero R. (2020). Classification of PPMI MRI scans with voxel-based morphometry and machine learning to assist in the diagnosis of Parkinson’s disease. Comput. Methods Programs Biomed..

[12] A Little M., E McSharry P., Roberts S.J., Costello D.A., Moroz I.M. (2007). Exploiting Nonlinear Recurrence and Fractal Scaling Properties for Voice Disorder Detection. Biomed. Eng. Online.

[13] Little M.A., McSharry P.E., Hunter E., Spielman J., Ramig L.O. (2008). Suitability of Dysphonia Measurements for Telemonitoring of Parkinson’s Disease. IEEE Trans. Biomed. Eng..

[14] Chawla N.V., Bowyer K.W., Hall L.O., Kegelmeyer W.P. (2002). SMOTE: Synthetic Minority Over-sampling Technique. J. Artif. Intell. Res..

[15] Gregorutti B., Michel B., Saint-Pierre P. (2016). Correlation and variable importance in random forests. Stat. Comput..

[16] Arif S., Wang J., Hassan T.U., Fei Z. (2019). 3D-CNN-Based Fused Feature Maps with LSTM Applied to Action Recognition. Future Internet.

[17] Ruby A.U., Theerthagiri P., Jacob I.J., Vamsidhar Y. (2020). Binary cross entropy with deep learning technique for Image classification. Int. J. Adv. Trends Comput. Sci. Eng..

[18] Kingma D.P., Ba J. (2015). Adam: A Method for Stochastic Optimization. arXiv.

[19] Ying X. (2019). An Overview of Overfitting and its Solutions. J. Phys. Conf. Ser..

[20] Mei J., Desrosiers C., Frasnelli J. (2021). Machine Learning for the Diagnosis of Parkinson’s Disease: A Review of Literature. Front. Aging Neurosci..

[21] Senturk Z.K. (2020). Early diagnosis of Parkinson’s disease using machine learning algorithms. Med. Hypotheses.

[22] Despotovic V., Skovranek T., Schommer C. (2020). Speech Based Estimation of Parkinson’s Disease Using Gaussian Processes and Automatic Relevance Determination. Neurocomputing.

[23] Wang W., Lee J., Harrou F., Sun Y. (2020). Early Detection of Parkinson’s Disease Using Deep Learning and Machine Learning. IEEE Access.

[24] Grover S., Bhartia S., Akshama, Yadav A., Seeja K.R. (2018). Predicting Severity Of Parkinson’s Disease Using Deep Learning. Procedia Comput. Sci..

